# *Rhodiola crenulata* Extract Alleviates Hypoxic Pulmonary Edema in Rats

**DOI:** 10.1155/2013/718739

**Published:** 2013-04-28

**Authors:** Shih-Yu Lee, Min-Hui Li, Li-Shian Shi, Hsin Chu, Cheng-Wen Ho, Tsu-Chung Chang

**Affiliations:** ^1^Graduate Institute of Medical Sciences, National Defense Medical Center, Taipei 114, Taiwan; ^2^Institute of Aerospace and Undersea Medicine, National Defense Medical Center, Taipei 114, Taiwan; ^3^Department of Biotechnology, National Formosa University, Yunlin 632, Taiwan; ^4^Department of Health and Nutrition Biotechnology, Asia University, Taichung 413, Taiwan; ^5^Department of Biochemistry, National Defense Medical Center, P.O. Box 90048-501, Nei-hu, Taipei 114, Taiwan

## Abstract

Sudden exposure of nonacclimatized individuals to high altitude can easily lead to high altitude illnesses. High altitude pulmonary edema (HAPE) is the most lethal form of high altitude illness. The present study was designed to investigate the ability of *Rhodiola crenulata* extract (RCE), an herbal medicine traditionally used as an antiacute mountain sickness remedy, to attenuate hypoxia-induced pulmonary injury. Exposure of animals to hypobaric hypoxia led to a significant increase in pathological indicators for pulmonary edema, including the lung water content, disruption of the alveolar-capillary barrier, and protein-rich fluid in the lungs. In addition, hypobaric hypoxia also increased oxidative stress markers, including (ROS) production, (MDA) level, and (MPO) activity. Furthermore, overexpression of plasma (ET-1), (VEGF) in (BALF), and (HIF-1**α**) in lung tissue was also found. However, pretreatment with RCE relieved the HAPE findings by curtailing all of the hypoxia-induced lung injury parameters. These findings suggest that RCE confers effective protection for maintaining the integrity of the alveolar-capillary barrier by alleviating the elevated ET-1 and VEGF levels; it does so by reducing hypoxia-induced oxidative stress. Our results offer substantial evidence to support arguments in favor of traditional applications of *Rhodiola crenulata* for antihigh altitude illness.

## 1. Introduction

The increase in tourism results in the exposure of more individuals, such as mountaineers, trekkers, and outdoor enthusiasts, to high altitude hypobaric hypoxia. Abruptly relocating nonacclimatized individuals to a high altitude can easily lead to a high altitude illness such as high altitude pulmonary edema (HAPE). HAPE, the most lethal form of high altitude illness [1], arises from a sustained lack of balance between the fluid into and out of the alveola. Clinical manifestations of HAPE include dyspnea at rest, cough, chest tightness, a wheezing sound, and frothy pink sputum. Proteinous fluid in the bronchoalveolar lavage fluid (BALF) of patients with a normal level of inflammatory cytokines was also found in the early phases of HAPE. Although the exact pathological mechanism of HAPE requires further elucidation, most studies concur that hypoxia-induced pulmonary hypertension, increased pulmonary capillary wall permeability, and impaired pulmonary transepithelial sodium transport all play central roles in HAPE progression [2, 3]. 

Preventive medicines for HAPE are currently limited in their applications, due to their adverse effects. For example, nifedipine can cause reflex tachycardia and peripheral edema; salmeterol causes tachycardia and is not suitable for heart disease patients; tadalafil is associated with headache and anorexia problems; and dexamethasone is associated with mood disorder and hyperglycemia [4]. Thus, a safer and more effective solution for the prevention of HAPE is definitely needed. 

Rhodiola, a traditional medicine in Asian countries, grows in cold, hypoxic, and radioactive environments at high altitudes, has been used for anti acute mountain sickness, and is regarded as an “adaptogen” in Tibet. Moreover in Asia, Rhodiola is also commonly used in eastern Europe for antidepression, antifatigue, and increasing work efficiency [5]. Rhodiola demonstrated an anti-hypoxia property as it significantly extended the survival time of mice exposed to hypoxia [6]. Recent studies also indicated that Rhodiola exerts anti-inflammatory [7], antioxidative, and ROS scavenger properties [8]. Furthermore, Rhodiola is generally considered to be a safe, non-toxic herbal medicine with no acute and subacute toxicity, based on long-term research with laboratory rats [5, 9]. Based on these findings, it is expected that Rhodiola may alleviate the pulmonary edema induced by high altitude hypobaric hypoxia. However, it is not known whether *Rhodiola crenulata* exerts beneficial effects in HAPE. The present study is designed to utilize a simulated hypobaric hypoxia rat model to investigate the efficacy of *Rhodiola crenulata* against HAPE.

## 2. Materials and Methods

### 2.1. Plant Material and Extraction Preparation

*Rhodiola crenulata* extract (RCE) was prepared by the Natural Products Research laboratory of the Department of Biotechnology at the National Formosa University, Taiwan. Briefly, the dry powder (1.0 kg) was extracted with 95% EtOH (4 × 10 L) and concentrated to yield RCE (242 g).

### 2.2. Animals

The 4–6-week-old male Sprague-Dawley rats weighing 150–180 g were purchased from BioLASCO Taiwan Co., Ltd. The rats were fed with regular food and water ad libitum and maintained with half and half light and dark cycles at 25 ± 0.5°C in the National Defense Medical Center's Laboratory Animal Center. The rats were divided into 6 groups (*n* = 6 in each group), including a control group (without hypoxia challenge), an RCE group (100 mg RCE without hypoxia), a hypoxia group, a hypoxia plus acetazolamide group (100 mg/kg), and hypoxia plus RCE groups (50 and 100 mg/kg). All animals were sacrificed after the 9-hour experiment. The experimental protocol was approved by the Institutional Animal Care and Use Committee of the National Defense Medical Center (IACUC-11-055). 

### 2.3. Hypobaric Hypoxia Exposure

The method was as described previously [10] with minor modifications on simulated altitude and hypoxic duration. Briefly, the rats were exposed to 8000 m in a simulated hypobaric hypoxia chamber at room temperature for 9 h. The rats were gavaged with RCE by a gastric cannula once daily for three days before exposure to hypoxia. The same steps were followed for the normoxic control group (saline) and acetazolamide group. The acetazolamide group was regarded as a positive control [11]. After hypoxic exposure, rats were anesthetized with sodium pentobarbital intraperitoneally (10 mg/kg); a tracheal cannula was then inserted, and the abdomen was opened. A blood sample was taken from the right ventricle. Finally, the lung was dissected.

### 2.4. Wet/Dry Weight Ratio

Lung wet/dry weight ratio was determined as described previously [12]. Briefly, after hypoxic exposure, the right lower lung lobe weight was measured immediately as wet weight and subsequently dried in an oven at 60°C for 72 h and then measured as dry weight.

### 2.5. Protein and Cytokine Concentration in BALF

Protein and cytokine concentrations were measured by the method previously described [12]. Briefly, BALF was obtained after hypoxic exposure by rinsing the left lung with saline twice (2 × 2.5 mL) and centrifuging it at 800 g for 10 min. The collected supernatant was measured for protein concentration using BCA protein assay reagents (Pierce, Rockford, IL, USA). The expression levels of tumor necrosis factor *α* (TNF-*α*), interleukin 1 *β* (IL-1*β*), VEGF, and cytokine-induced neutrophil chemoattractant 1 (CINC-1) were performed using ELISA kits (R&D Systems Inc., Minneapolis, MN, USA) and following the manufacturer's instructions.

### 2.6. Malondialdehyde (MDA) Level in Lung Tissue

The MDA level in the lung tissue was quantified by a commercial kit (Cayman Chemical Co., Ann Arbor, MI) as described previously [12]. The right upper lung lobe was homogenized in RIPA buffer with protease inhibitors. After being centrifuged at 1600 g for 10 min, an aliquot (100 *μ*L) of the supernatant was mixed with 100 *μ*L SDS and 4 mL color reagent containing 8.1% thiobarbituric acid. Samples were then placed in boiling water for 1 h and centrifuged at 1600 g for 10 min. The supernatant was collected, and its absorbance was measured at 532 nm.

### 2.7. Myeloperoxidase (MPO) Activity in Lung Tissue

MPO activity was determined as described [12] previously. Briefly, the right upper lung lobe was sonicated and freeze-thawed for three cycles in 50 mM KH_2_PO_4_ buffer. Homogenates were then centrifuged at 14000 rpm for 20 min. A 10 *μ*L of supernatant was reacted with 190 *μ*L assay buffer containing 1.68 mM 3,3′,5,5′-Tetramethylbenzidine (TMB) and 0.00015% hydrogen peroxide. The decomposition rate of hydrogen peroxide was determined by measuring the TMB oxidative product at 450 nm.

### 2.8. ROS Level in Lung Tissue

The ROS level was determined using DCFH-DA (2,7,dichlorofluorescein diacetate) measured by spectrofluorometer [13]. Briefly, lung tissue was homogenated and centrifuged at 14000 rpm for 20 min. The supernatant was incubated with 10 mM DCFH-DA in PBS at 37°C for 15 min and then measured at an excitation and emission wavelength of 485 and 538 nm, respectively. Data were normalized by the sample protein concentration.

### 2.9. Endothelin-1 (ET-1) Level in the Plasma

After hypoxic exposure, the blood samples were taken from the right ventricle after the thoracotomy and centrifuged at 1000 g at 4°C for 20 min as described previously [12]. The ET-1 level of the plasma samples was assayed using a commercial kit (R&D Systems Inc., Minneapolis, MN, USA) in accordance with the manufacturer's instructions.

### 2.10. Lung Histology

A lung histological examination was performed by the method previously described [12]. Briefly, the lung was perfused with 10% formaldehyde through the trachea and soaked in 10% formaldehyde solution for 24 h. The lung histology was determined by hematoxylin and eosin stains. 

### 2.11. Western Blot Analysis for HIF-1 *α*

HIF-1 *α* in the lung tissue was quantified as described [14] previously. Briefly, lung tissue was homogenized in RIPA buffer with protease inhibitors. Equal amounts of protein in each sample were separated by sodium dodecyl sulfate (SDS), 10% polyacrylamide gel electrophoresis (PAGE), and transblotted onto polyvinylidene difluoride (PVDF) membranes (Millipore, Bedford, MA). Immunoblotting was performed with antibodies for HIF-1 *α* (Millipore, Bedford, MA) and *β*-actin (Chemicon, Temecula, CA). Signals were visualized with an enhanced chemiluminescence kit (ECL, Amersham, UK), followed by exposure to X-ray films.

### 2.12. Statistical Analysis

All of the data represent mean ± S.E.M. Statistical difference among group means was determined with one-way ANOVA with repeated measures, followed by using Newman-Keuls test with SPSS 14.0 for Windows software. *P* < 0.05 was considered to be statistically significant.

## 3. Results

### 3.1. RCE Suppresses Hypoxia-Induced Pulmonary Edema and Alveolar Extravascular Protein

**Figure 1:**
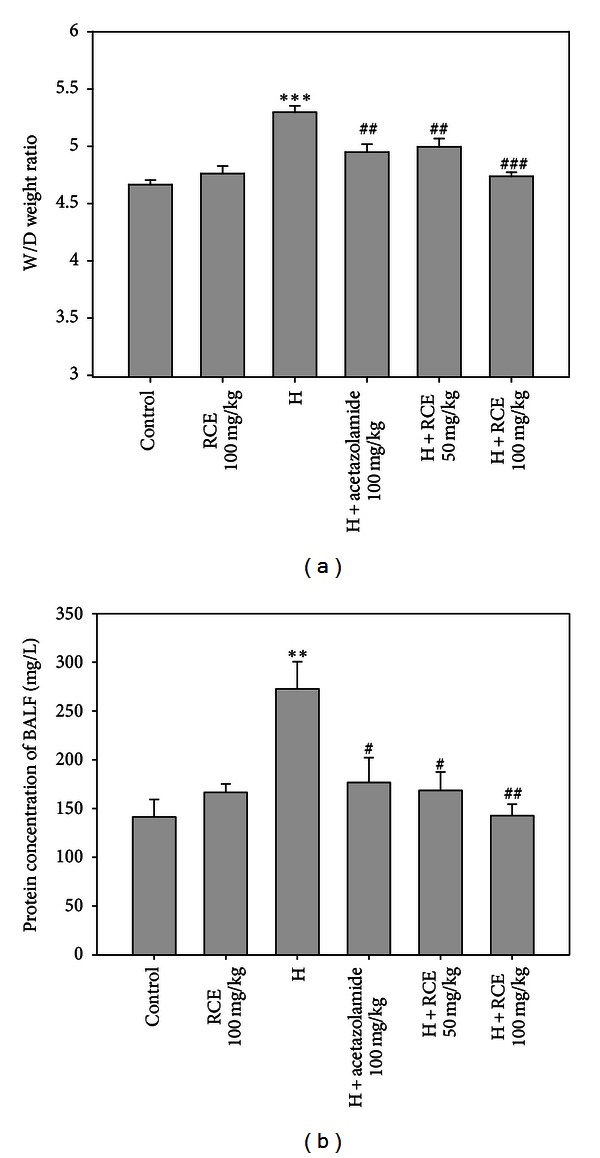
RCE significantly attenuated hypoxia-induced pulmonary edema and alveolar extravascular protein. Effect of RCE on (a) W/D weight ratio and (b) BALF protein concentration. The rat control groups were treated with saline (control) or RCE under normoxia. The rat hypoxia groups received hypoxia treatment alone (H) or in combination with different doses of RCE (H + RCE). Results represent mean ± SEM (*n* = 6). *P < 0.05; **P < 0.01; ***P < 0.001 versus control; ^#^ *P* < 0.05; ^##^ *P* < 0.01; ^###^ *P* < 0.001  versus hypoxia (H).

Pulmonary edema was assessed by examining the wet/dry (W/D) weight ratio and BALF protein concentration. As shown in Figure 1(a), exposure to simulated hypobaric hypoxia caused a significant increase in lung W/D weight ratio (5.30 ± 0.06, *P* < 0.001) compared to that in the control group (4.67 ± 0.04). This hypoxia-induced gain in wet/dry weight ratio was significantly suppressed in the RCE groups (5.00 ± 0.07 and 4.74 ± 0.04 for 50 and 100 mg/kg of RCE, resp., *P* < 0.01 and *P* < 0.001). No significant difference was observed between the normoxic RCE and saline control groups. Results shown in Figure 1(b) indicate that a significantly higher BALF protein concentration was also observed in hypoxia rats (272.8 ± 27.8 mg/L, *P* < 0.01) compared with normoxic rats (141.4 ± 17.9 mg/L). Treatment with RCE effectively attenuated the protein concentration in BALF compared with the hypoxia group. Strong efficacy was observed with the RCE concentration at the dose of 100 mg/kg (142.8 ± 11.6 mg/L,  *P* < 0.001). As an experimental control, acetazolamide exhibited significant efficacy in suppressing the hypoxia-induced lung W/D weight ratio and BALF protein concentration. 

### 3.2. Hypoxia Confers No Significant Effect on Inflammatory Cytokine Levels in the Hypoxia-Treated Lung

**Figure 2:**
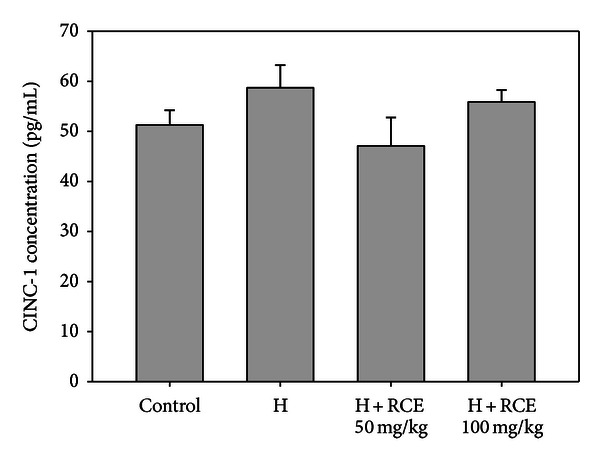
Hypoxia exhibits no significant effect on the level of CINC-1. Results represent mean ± SEM (*n* = 6).

In the present study, exposure to hypoxia only induced a slight increase in the CINC-1 (58.0 ± 4.6 pg/mL) level, which does not differ significantly when compared with the normoxic group (51.2 ± 2.9 pg/mL). Similarly, there is also no significant difference in the BALF CINC-1 level in the RCE-treated groups (47.2 ± 5.7 and 55.9 ± 2.4 pg/mL for 50 and 100 mg/kg of RCE, resp.) compared to the hypoxic group (Figure 2). The BALF TNF-*α* and IL-1*β* levels were also quantified; however, proinflammatory cytokines were not detectable under our experimental conditions (less than 5.0 pg/mL).

### 3.3. RCE Attenuates Oxidative Stress Markers in the Lung with Hypoxia Insults

**Figure 3:**
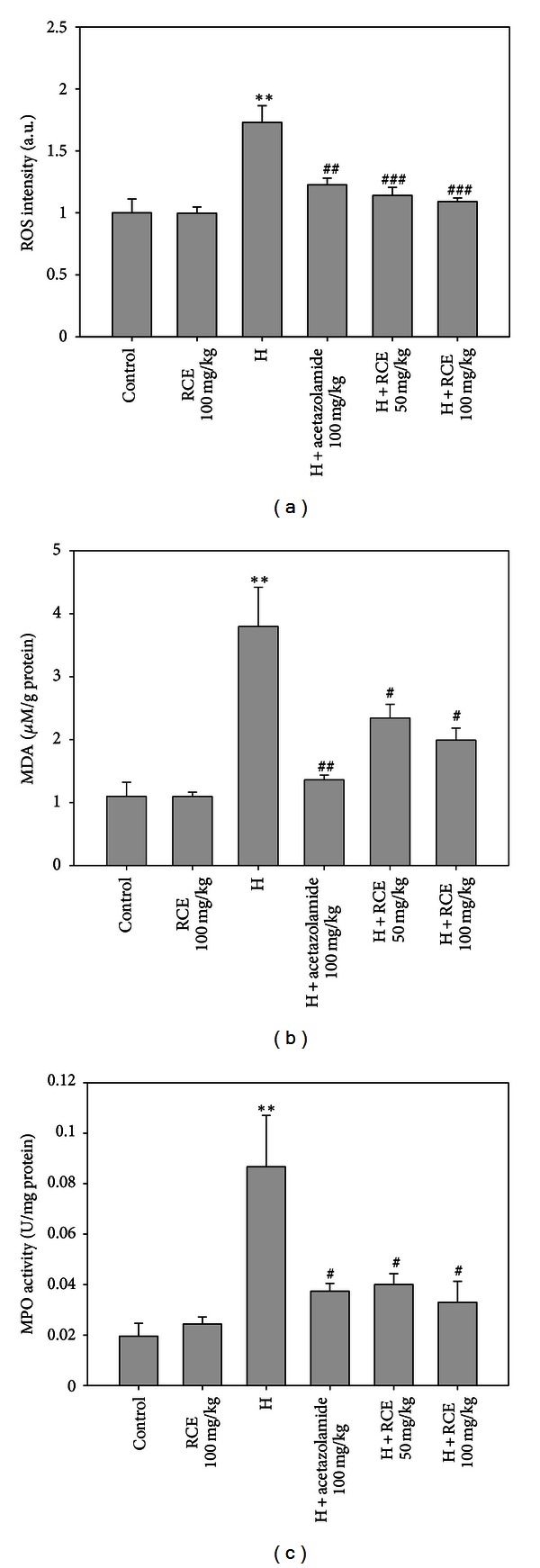
Effects of RCE on oxidative stress markers in lung tissue. RCE significantly attenuates (a) hypoxia-induced ROS bursts, (b) lipid peroxidation, and (c) the MPO activity in lung tissue. Results represent mean ± SEM (*n* = 6). *P < 0.05; **P < 0.01; ***P < 0.001 versus control; ^#^ *P* < 0.05; ^##^ *P* < 0.01; ^###^ *P* < 0.001 versus hypoxia (H).

The protective effect of RCE on hypoxia-induced oxidative stress was evaluated by measuring the ROS and MDA levels and the MPO activity. Results demonstrate a significantly elevated ROS level in lung tissue upon hypoxia exposure (1.73 ± 0.21-fold, *P* < 0.01) (Figure 3(a)). Pretreatment with RCE significantly curtailed the hypoxia-induced ROS burden in a dose-dependent manner to the normoxic control group (1.09 ± 0.03-fold at the dose of 100 mg/kg, *P* < 0.001). Similarly, the MDA level in lung tissue was also significantly increased in the hypobaric hypoxia group (3.8 ± 0.7 *μ*M/g protein, 3.5 fold, *P* < 0.01) compared with the normoxic group (1.1 ± 0.2  *μ*M/g protein). Both RCE groups (50 and 100 mg/kg) significantly curtailed the hypoxia-induced MDA levels in the lung tissue (2.3 ± 0.2 and 2.1 ± 0.2 *μ*M/g protein, resp., *P* < 0.05) (Figure 3(b)). The measurement of MPO also serves as a reliable marker for oxidative stress in various tissues [15]. As shown in Figure 3(c), MPO activity in lung tissues increased dramatically (0.087 ± 0.020 U/mg protein, 4.3-fold, *P* < 0.01) in the hypoxia group compared with the control (0.020 ± 0.005 U/mg protein). Pretreatment with both 50 and 100 mg/kg of RCE significantly reduced the hypoxia-induced MPO activity (0.040 ± 0.004 and 0.033 ± 0.008 U/mg protein, resp., *P* < 0.05). RCE alone showed no significant effect on these oxidative stress markers.

### 3.4. RCE Reduces the ET-1, VEGF, and HIF-1*α* Levels in the Hypoxia-Injured Lung

**Figure 4:**
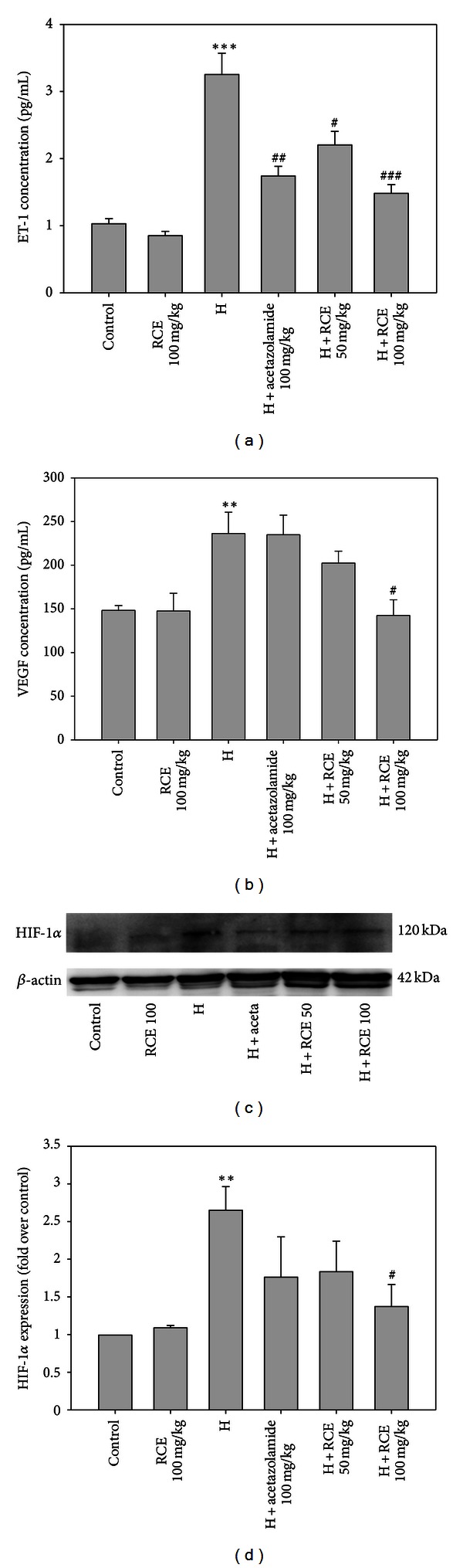
RCE inhibited plasma ET-1, BALF VEGF, and HIF-1*α* expressions in hypoxia-treated animals. Hypoxia increased and RCE attenuated (a) plasma ET-1 level (*n* = 6), (b) BALF VEGF level (*n* = 6), and (c) HIF-1*α* protein expression level (*n* = 3). (d) Quantitative analysis of the relative level of HIF-1*α* from (c). Results represent mean ± SEM. *P < 0.05; **P < 0.01; ***P < 0.001 versus control; ^#^ *P* < 0.05; ^##^ *P* < 0.01; ^###^ *P* < 0.001  versus hypoxia (H).

As shown in Figure 4(a), a more than 3-fold increase in plasma ET-1 level was found in animals that were exposed to hypobaric hypoxia (3.3 ± 0.3 pg/mL, *P* < 0.001). Pretreatment with RCE (50 and 100 mg/kg) significantly attenuated the hypoxia-induced ET-1 level dose dependently (2.2 ± 0.2 and 1.5 ± 0.1 pg/mL, with *P* < 0.05 and *P* < 0.001, resp.). The RCE at 100 mg/kg exhibited better efficacy than the acetazolamide group in alleviating the plasma ET-1 level. In addition to plasma ET-1, the VEGF level in BALF was also examined for lung injury. The BALF VEGF level demonstrated a significant increase in the hypobaric hypoxia group (236.3 ± 24.4 pg/mL, *P* < 0.01) compared to that of the control group (148.3 ± 5.5 pg/mL). Pretreatment with 100 mg/kg of RCE significantly ameliorated the hypobaric hypoxia-induced BALF VEGF to a level which showed no significant difference from the normoxic group (142.5 ± 18.0 pg/mL, *P* < 0.05) (Figure 4(b)). Consistent with the plasma ET-1 and BALF VEGF results, the expression level of the HIF-1*α* protein was noticeably upregulated in the hypoxia group (2.7 fold, *P* < 0.01). Pretreatment with 100 mg/kg of RCE caused a significant decrease in hypoxia-induced HIF-1*α* overexpression (1.4-fold over control, *P* < 0.05). It should be noted that the 50 mg/kg RCE group also suppressed the hypoxia-induced VEGF and HIF-1*α* levels, though not significantly.

### 3.5. RCE Confers a Protective Effect on the Lung with Hypoxia Injury

**Figure 5:**
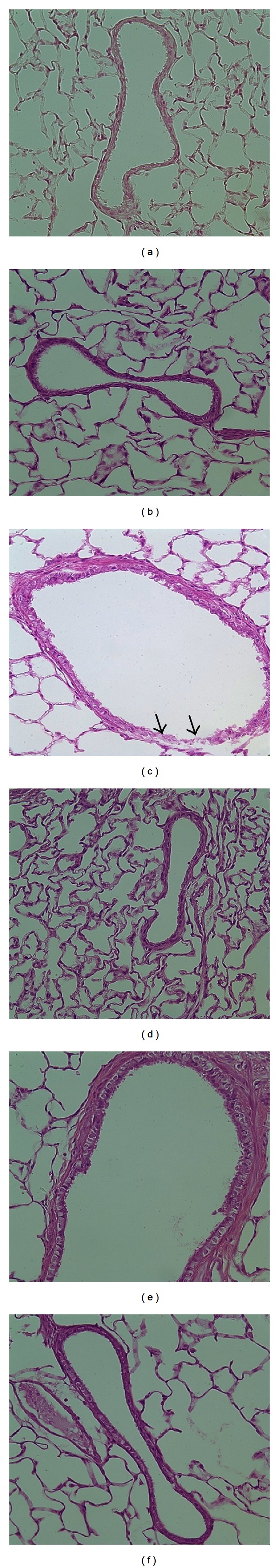
Lung histology. Histological examination was performed by photomicrography (original magnification ×400). Exposure to hypoxia led to a disruption of the alveolar-capillary barrier as indicated by the arrow and congested vascular wall. Treatment with RCE maintained the integrity of the alveolar-capillary barrier from hypoxic insults. (a) Control, (b) RCE, 100 mg/kg, (c) hypoxia (H), (d) H + acetazolamide, 100 mg/kg, (e) H + RCE, 50 mg/kg, and (f) H + RCE, 100 mg/kg. Pictures depicted here represent three independent experiments.

The histological examination was used to evaluate the protective effect of RCE to the hypoxia-induced lung injuries. The normoxic group showed normal alveolar-capillary barrier with no neutrophil and red cell filtration. However, the changes in septal thickening, escaped red cells, neutrophil filtration in alveola, and disruption of alveolar-capillary (as the arrow indicated) and severely congested vascular wall were evident in the hypoxia group. Both RCE and acetazolamide significantly attenuated the changes in lung histology in comparison with the hypoxia group. These results demonstrated that RCE confers marked efficacy in maintaining the integrity of alveolar-capillary barrier from hypoxic insults. There is no difference between the normoxic groups with or without RCE treatment (Figure 5).

## 4. Discussion

People who engage in physical activities at high altitudes, such as athletes, mountaineers, and military service personnel, frequently experience hypoxic stress. It is thus critically important for them to keep in normal physiological condition in order to avoid the potentially serious health consequences of high altitude illnesses such as HAPE. Acetazolamide, a diuretic approved only by the U.S. Food and Drug Administration as a drug for anti acute mountain sickness [6], served as the positive control in this study. Although acetazolamide is a mild diuretic, its action has more to do with it being a respiratory stimulant because it causes a metabolic acidosis. Our results showed that it is an effective drug for the prevention of hypoxia-induced lung injuries. However, acetazolamide has undesirable adverse effects that seriously limit its applications, including a polyuria-like symptom, metabolic acidosis, and gastrointestinal disturbances. Therapeutic approaches with natural products such as Rhodiola would be a promising candidate to prevent the environmental impacts of high altitude conditions.

In an attempt to evaluate the efficacy and mechanism of Rhodiola in prevention of hypobaric hypoxia induced lung injury, an altitude of 8000 meters was simulated using a simulated hypobaric hypoxia system to induce HAPE-like syndrome. Markers for pulmonary edema, oxidative stress and pulmonary hypertension and vascular permeability were assessed under hypobaric hypoxia in the presence of RCE. In this study, we showed that when rats were exposed to these simulated conditions, it led to significant increase in lung water content, protein-rich alveolar fluid, higher MPO activity, ROS burden, and lipid peroxidation in lung tissue, as was observed in HAPE. In addition, the plasma ET-1, VEGF in BALF and HIF-1*α* levels were also increased significantly in lung tissue. Meanwhile, we also observed the disruption and swelling of the alveolar-capillary barrier through the histological examination. However, administration of RCE significantly suppressed all the pulmonary edema indicators. These results indicated that RCE is able to attenuate HAPE-like syndrome under acute hypoxic circumstances. 

It was reported that hypoxia-induced oxidative stress is associated with pulmonary hypertension and vascular leakage, which largely contributed to alveolar-capillary barrier dysfunction [16–18]. However, antioxidant agents attenuted not only oxidative stress, but also pulmonary hypertension and vascular leakage [19, 20]. These reports suggested that decreasing the hypoxia-induced burden of oxidative stress may curtail the manifestations of HAPE. In the present study, we showed that RCE alleviates hypoxia-induced oxidative stress markers, including lipid peroxidation, MPO activity, and ROS bursts. Consistently, Rhodiola has been shown to exhibit antioxidant and ROS scavenger activities in different cultured cell lines and animal models [5, 8]. Thus, it is very likely that the efficacy of RCE in our study correlates with the reduction of hypoxic oxidative stress in the hypobaric hypoxia animals. 

 ET-1 is a potent and long lasting vasopressive peptide which is present in abundance in the lungs. An increase in ET-1 is associated with the pathological progression of HAPE and severity of pulmonary hypertension in humans, as it was shown that the ET-1 receptor antagonist proved to be efficacious against pulmonary hypertension [16, 21]. These findings suggest that plasma ET-1 level is a marker of pulmonary hypertension, which play important roles in the pathological progression of HAPE. In this study, we showed that RCE remarkably reduced the hypoxia-induced plasma ET-1 levels in a dose-dependent manner. The findings suggest that RCE attenauated hypoxia-induced pulmonary hypertension and vasoconstriction.

Although hypoxia-induced pulmonary hypertension is an etiologic factor in the pathological progression of HAPE, other cofactors such as VEGF are still required to trigger HAPE [22, 23]. In fact, exposure to hypoxia causes VEGF overexpression [17]. In addition to induction by hypoxia, ET-1 also causes an exaggerated rise in capillary hydrostatic pressure; it does so by directly increasing microvascular permeability through the induction of VEGF overexpression [24]. Overexpression of VEGF in the lung was shown to cause vascular leakage and pulmonary edema in mice [18]. By contrast, VEGFR-1, which can function as a decoy receptor for VEGF, dampened the severity of pulmonary edema [18]. These findings strongly suggest that the alveolar VEGF level also shares a strong association with the pathogenesis of HAPE. In the present study, we showed that RCE effectively reduced the BALF VEGF level under hypoxic condition. The results suggest that RCE alleviated the pulmonary edema partially via maintenance of vascular permeability.

In the case of ET-1 and VEGF, it is known that both of these molecules contributed to alveolar-capillary barrier dysfunction by various mechanisms [16–18]. Combined with a histological examination, we further demonstrated that RCE effectively attenuated hypoxia-induced disruption of the alveolar-capillary barrier. The findings are consistent with those of similar studies in that decreasing the disruption of alveolar-capillary barrier and the vascular Leakage is able to reduce HAPE syndrome [10, 25]. In addition, the effect of overexpressed ET-1 and VEGF might be linked with oxidative stress in hypobaric hypoxia animals, as it was shown that the ROS to HIF-1*α* signaling pathway is involved in both hypoxia-induced ET-1 and VEGF overexpression [20, 26]. Furthermore, we also showed that RCE suppressed hypoxia-induced HIF-1*α* accumulation. As the RCE effectively decreased oxidative stress and HIF-1*α* overexpression in our study, we suggest that RCE suppresses hypoxic ET-1 and VEGF overexpression via inhibition of ROS-HIF pathway. 

The roles of proinflammatory cytokines in the rodent models of HAPE are controversial. Recent studies indicate that the proinflammatory cytokines play a role in similar rodent models of HAPE [10, 13]. Our data, however, indicated that levels of proinflammatory cytokines, such as TNF-*α* and IL-1, in BALF were undetectable among all groups in rat models between normoxia and hypoxia groups. The level of CINC-1, a chemokine whose upexpression is dependent on the NF*κ*B signaling pathway [27], also showed no significant difference. Thus, proinflammatory cytokines may not play a significant role in the animal model of HAPE. These observations are consistent with the previous findings that showed extremely low concentrations in the level of BALF TNF-*α* and IL-1 and no significant difference between HAPE patients and normal patients [3]. Thus, our results further demonstrated that inflammatory cytokines do not mediate an early event of hypobaric hypoxia-induced pulmonary edema. Furthermore, these findings also confirm the notion that inflammation is a result, rather than a causative factor, of HAPE [3]. Our results also suggest the early phase of hypoxia-induced pulmonary hypertension and increased vascular permeability as the causes of HAPE, and that subsequently leads to alveolar-capillary barrier dysfunction and pulmonary edema. This scenario may also clarify the finding that HAPE symptoms appear in 2–4 days after exposure to high altitude. Taken together, our results indicate that the RCE exerts its efficacy by suppression of the early phase of pathological progression of HAPE.

Genus Rhodiola (Crassulaceae) contains about 96 species worldwide and has been well known for its excellent antioxidant activities. Up to now, most studies focus on the efficacy of *Rhodiola rosea*, which has been a valuable medicinal plant in European countries for thousands of years [5]. Although Rhodiola species may have certain similarities, they are different in terms of their content of bioactive components and their traditional applications in regional indigenous remedies. Among them is *Rhodiola crenulata*, which is regarded as the best in quality and is included in the Pharmacopoeia of China [28] and has been used as an antiacute mountain sickness herbal medicine in Tibet since ancient times. However, a detailed study demonstrating the potential protective effect of *Rhodiola crenulata* on hypobaric hypoxia-induced pulmonary edema is still lacking. In the study, we present evidence to demonstrate that a popular folk medicine, RCE, exhibited comparable or superior efficacy to acetazolamide in the prevention of hypoxia-induced lung injury. Although RCE confers protective effects on many indicators of hypoxia induced pulmonary edema, the mechanism of the protective effect of RCE in pulmonary edema still requires further elucidation. For example, impaired pulmonary transepithelial sodium transport was reportedly involved in the pathological progression of HAPE [29]. Thus, it is interesting to examine whether RCE is efficacious regarding pulmonary sodium transport. It is also important to evaluate the contributory and functional role played by salidroside, the main bioactive compound in RCE, in the protective effects of Rhodiola in HAPE. The identification of additional active protective components and expansion of the potential applications of RCE warrant further study. In spite of this, the present study was carried out with some limitations, such as small numbers of rats in each group and the extreme simulated altitude used. More rats are definitely required to verify our findings for future clinical applications of Rhodiola. The extreme simulated altitude used here is necessary to consistently induce HAPE-like syndrome as other similar studies described previously [10, 13, 25]. It should also be noted that we did not measure the pulmonary pressure directly, owing to the limitations of a simulated hypoxia chamber.

**Figure 6:**
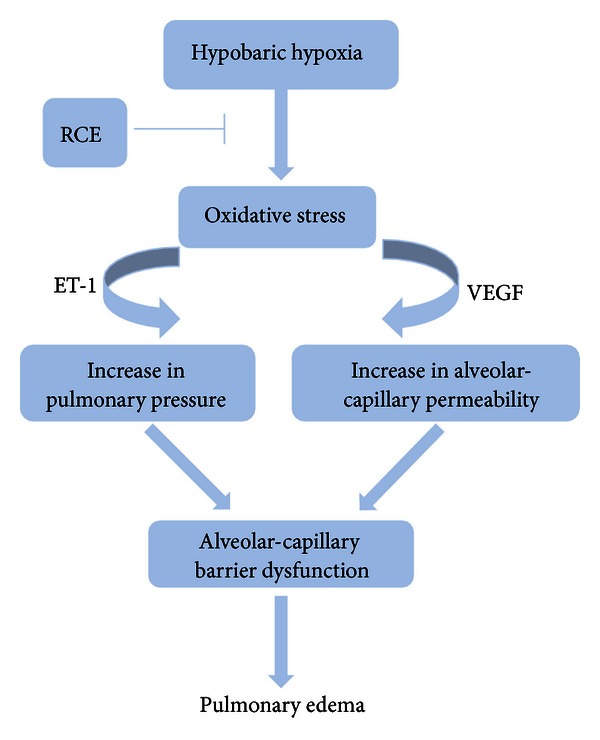
The proposed mechanism of RCE for alleviating the pulmonary edema. RCE attenuates the hypoxia-induced alveolar-capillary barrier dysfunction by reducing oxidative stress.

 In conclusion, we present evidence to show that RCE exhibits remarkable antioxidative stress ability and reduces alveolar-capillary barrier dysfunction to alleviate pulmonary edema induced by hypobaric hypoxia (Figure 6). Thus, Our findings showed that RCE may be helpful for the prevention of HAPE and provide evidence to support traditional applications of *Rhodiola crenulata* for anti high altitude illness.
